# Profound Differences in Virus Population Genetics Correspond to Protection from CD4 Decline Resulting from Feline Lentivirus Coinfection

**DOI:** 10.3390/v2122663

**Published:** 2010-12-10

**Authors:** Abinash Padhi, Howard Ross, Julie Terwee, Sue VandeWoude, Mary Poss

**Affiliations:** 1 Department of Biology, The Pennsylvania State University, University Park, PA 16802, USA; E-Mail: aup17@psu.edu; 2 School of Biological Sciences, University of Auckland, Auckland, 1142, New Zealand; E-Mail: h.ross@auckland.ac.nz; 3 Department of Microbiology, Immunology, and Pathology, Colorado State University, Ft. Collins, CO 80523, USA; E-Mails: jat@lamar.colostate.edu (J.T.); Sue.Vandewoude@colostate.edu (S.V.); 4 Fogarty International Center, National Institutes of Health, Bethesda, MD 20892, USA; 5 208 Mueller Lab, Center for Infectious Disease Dynamics, The Pennsylvania State University, University Park, PA 16802, USA

**Keywords:** Lentivirus, dual infection, population genetics, genetic bottleneck, viral temporal dynamics, mutation rate

## Abstract

CD4 decline is a hallmark of disease onset in individuals infected with Feline Immunodeficiency Virus (FIV) or Human Immunodeficiency Virus type 1 (HIV-1). Cats that are infected with a poorly replicating, apathogenic FIV (PLV) prior to exposure to a virulent FIV strain (FIVC) maintain CD4 numbers by mechanisms that are not correlated with a measurable adaptive immune response or reduction in circulating viral load. We employed population genetic approaches based on the 3′ portion of the viral genome to estimate the population structure of FIVC from single and dual infected cats. In dual infected cats, FIVC effective population size was decreased during the initial viral expansion phase, and after three weeks of infection, the population declined sharply. The FIVC population recovered to pre-bottleneck levels approximately seven weeks post-FIVC infection. However, the population emerging from the bottleneck in dual infected cats was distinct based on estimates of temporal population structure and substitution profiles. The transition to transversion rate ratio (κ) increased from early to late phases in dual infected cats due primarily to a decrease in transversions whereas in single infected cats, κ declined over time. Although one clone with extensive G to A substitutions, indicative of host cytidine deaminase editing, was recovered from a dual infected cat during the bottleneck, the post bottleneck population had an overall reduction in G to A substitutions. These data are consistent with a model of PLV-induced host restriction, putatively involving host DNA editing, that alters the dynamics of FIVC throughout the course of infection leading to disease attenuation.

## 1. Introduction

Lentivirus infections cause profound changes in immune function reflected by alterations of circulating T cell phenotype, most notably decline of CD4 T cells, in infected felines and humans. To date, the most effective and widely used therapeutic intervention strategies aim to reduce viral replication with specific inhibitors of different parts of the virus life cycle; immunological correlates of protection against disease are poorly understood. There are intriguing reports that a preexisting virus infection can ameliorate disease when an individual becomes infected with a virulent lentivirus. For example, a primary HIV-2 infection delays the onset of AIDS in individuals who secondarily are infected with HIV-1 [1]. Hepatitis G virus has also been reported to attenuate HIV-1 infection *in vivo* and *in vitro* [2]. In both cases, the mechanisms of disease attenuation are unclear but do not appear to be solely due to host adaptive immunity.

The mechanism by which a preexisting apathogenic lentivirus infection protects against disease caused by a virulent lentivirus can be explored experimentally using cats infected with Feline Immunodeficiency Virus (FIV). Cats experimentally infected with a pathogenic molecular clone of FIV subtype C (FIVC36; FIVC hereafter) develop an AIDS-like syndrome that is very similar to that observed for human AIDS [3]. If cats were initially infected with an apathogenic lentivirus derived from a wild cougar, strain poorly replicating, apathogenic FIV (PLV), they did not experience CD4 decline and neutropenia when co-infected with FIVC [4,5]. As with HIV-1 coinfections, the altered outcome of infection did not appear to be due to host adaptive immunological responses.

In this report, we evaluated the effect of PLV on FIVC infection dynamics by analyzing virus population genetic structure. The null hypothesis is that if there is no effect of PLV on FIVC, genetic variability in FIVC should be similar in the single and dual infection environment. On the other hand, if our analyses indicate that different FIVC population genetics profiles exist in single and dual infections, the parameters that differ between the two populations will inform the mechanism of PLV-induced protection. The extent of genetic variations and associated factors can be evaluated by estimating several population genetic parameters. Whereas the census population size represents a total count of viral particles, the effective population size, N_e_ (θ = 2N_e_μ) [6] is implicitly associated with the mutation rate (μ) and represents the number of parental virions contributing genetic information to successive generations. Thus, differences in census and effective population size can inform important interactions that occur in complex coinfection systems such as the one under study. The viral effective population size is inversely associated with random genetic drift. Thus, the effect of genetic drift will be stronger on viruses with smaller effective population size and, as a consequence, the overall mutation rate in this population would be higher. Stochastic processes as defined by drift have been recognized as determinants that change the genetic makeup of several RNA viruses [7] including lentiviruses [8–11]. This is particularly important because, in a neutrally evolving population, genetic drift can reduce the overall genetic variation and, consequently, fitness in the population; a concept explained by Muller’s ratchet [12]. Muller’s ratchet predicts that genetic drift and associated higher mutation rate in a small asexual population will cause an excess of deleterious mutations to accumulate, which eventually leads to a genetic bottleneck. Although there is increasing evidence on the operation of Muller ratchet in RNA viruses [13–18], with few exceptions [19–21], evidence of Muller’s ratchet is limited to lentiviruses.

The main objective of the present study was to determine whether a virulent FIVC strain exhibited a genetic profile unique to the dual infection environment. We evaluated the rate of evolution, patterns of nucleotide substitution, population genetic structure, and population dynamics of FIVC from single and dual infected cats. Our results demonstrate that PLV infection impacts FIVC throughout the course of infection. These data are significant because they suggest that disease prevention induced by previous infection with an attenuated virus is more closely associated with changes in viral dynamics than to known correlates of host adaptive immunity.

## 2. Results and Discussion

### 2.1. FIVC Genetic Diversities and Population Dynamics Differ Significantly Between Single and Dual Infection Environments

**Figure 1:**
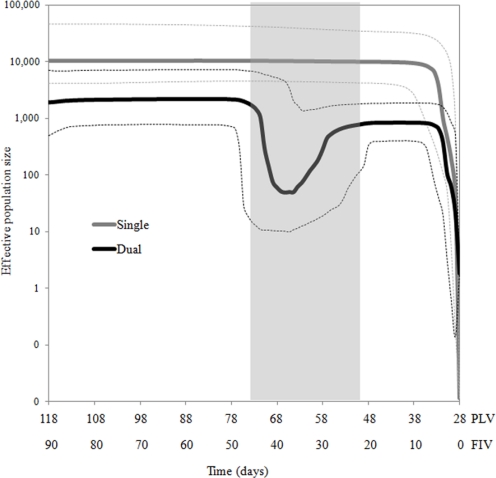
FIVC genomic diversity in dual and single infection. Bayesian skyline plot depicting dynamics of FIVC effective population size in single (grey) and dual (black) infections. Thick lines indicate median values and dotted lines indicate 95% confident intervals. The time scales are referenced to the day of PLV infection, and to the day of FIVC infection, which occurred 28 days after cats were infected with PLV. Shading highlights severe constriction of effective population size, indicative of a population bottleneck.

To determine the population genetic features of FIVC in single and dual infection environments, we estimated viral genetic diversity indices using distance-based summary statistics (Table S1). Tajima’s D for FIVC in single (D = −2.94749) and dual (D = −2.94989) infected groups were significantly less than zero (p < 0.001). This indicated that, compared to expectations for neutrally evolving populations, rare alleles were in excess in both single and dual infection environments and the populations were expanding or under purifying selection. We next performed Bayesian skyline analyses [22,23] because Tajima’s D estimates do not capture temporal dynamics of these viral populations. The mean coefficient of variation (CoV) of FIVC in single and dual infection environments was very high. In neither case did the lower 95% highest-posterior density (HPD) encompass zero (Table 1), which indicated that there were large scale rate heterogeneities among viral isolates in both infection environments and the populations were not exhibiting clock-like behavior. The mutation rate of FIVC in dual infected cats was higher than that of single infected cats and there was no overlapping of 95% HPD. The Bayesian skyline plot (BSP) revealed that both FIVC populations showed sudden expansion during the first week of infection (Figure 1). However, the effective population size of FIVC in dual infected cats was significantly smaller than the FIVC population in the single infection environment with no overlapping of 95% HPD. Further, while FIVC in the single infection environment remained stable for the duration of the experiment, there was a sharp population decline representing a bottleneck from day 21 to 45 post FIVC infection in FIVC from dual infected cats (Figure 1). The virus population recovered from this marked restriction in genetic diversity by approximately seven weeks post-infection and was stable thereafter. However, effective FIVC population size remained lower in dual *vs.* single infection (Figure 1).

### 2.2. Temporal Population Structure

**Figure 2:**
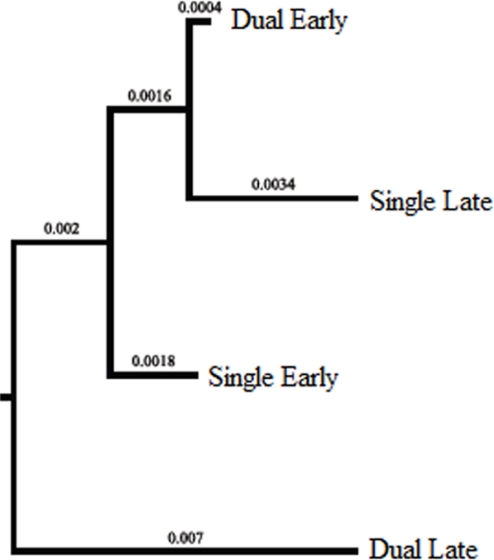
Clustering of FIVC from dual and single infections. F_ST_-based neighbor joining tree showing the relationships among the temporally-spaced infection groups. Branch lengths are shown at the base of the respective nodes.

The Bayesian skyline analysis suggested that the viral populations in each infection environment could be temporally structured. To investigate this possibility, the data were partitioned into four groups: single-early (SE), single-late (SL), dual-early (DE), and dual-late (DL) and the genetic variances were evaluated using analyses of molecular variance (AMOVA). The genetic variances attributed to among groups (single *vs.* dual) were not significant (Table 2), which was expected because total genetic variation was averaged across time points in this analysis. However, there was significant genetic differentiation between SE and SL, and between DE and DL (p < 0.001), indicating temporal structuring of FIVC populations in both single and dual infection. Further, pairwise F_ST_ analyses showed that DL was significantly different from the other groups (p < 0.0001). A neighbor-joining tree based on pairwise F_ST_ showed that DL was an out-group to the other three groups (Figure 2). Thus, the dual late FIVC population, which represents the population that recovered from a bottleneck event (Figure 1), showed unique population structuring.

### 2.3. Pattern of Nucleotide Substitution

The temporal and spatial genetic structure in a neutrally evolving population is presumably due to stochastic mutational processes. However, forces such as rapid adaptation to the locality by natural selection can contribute significantly to population genetic structure. We therefore investigated the nature of selection and mutational profile to enhance our understanding of the mechanisms leading to the unusual FIVC genetic structuring detected in dual infected cats. The overall dn/ds values for all protein coding genes ranged from 0.2 to 5.1 (95% HPD) (Table S2), but none of the codons in any gene were detected as positively selected. This supports our earlier findings (Table S1) that purifying selection was the major driving force in the evolution of FIVC in both infection environments.

Early in the course of infection, transitions, which typically cause synonymous changes, are more frequent than transversions, which more often cause a change in amino acid. We compared the transition to transversion rate ratio (kappa, κ) for the dUTPase/integrase portion of *pol (ui)*, *vif* and *env* of each of the four groups (Table 3). Our results revealed that κ was increased two- to three-fold in dual late compared to dual early FIVC genomes. In contrast, there was a reduction in κ from the early to late phase in FIVC from single infected cats (Table 3). There was a two- to four-fold increase in κ from FIVC in the dual late compared to single late infection environment.

**Figure 3:**
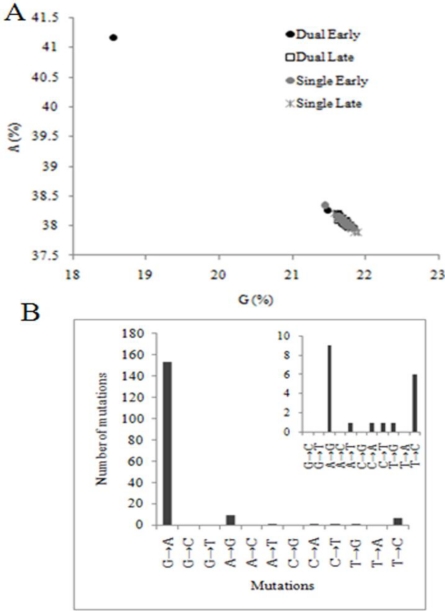
Nucleotide substitution frequency in a hypermutated clone from a dual infected cat. (**A**) Plot of percentage A *versus* G for all clones in all the four groups. The outlier clone, dF96B68_d45, is identified in the upper left quadrant. (**B**) Specific mutations noted in the hypermutated clone relative to parental virus. Inset indicates non-G to A transitions on an expanded scale.

**Figure 4:**
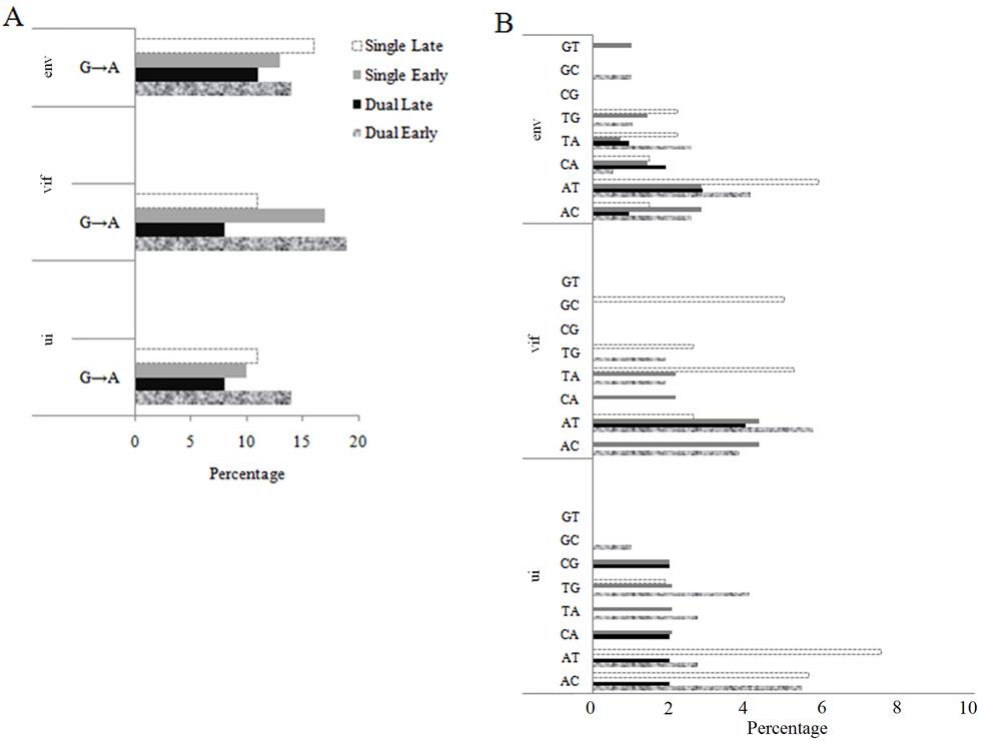
Percentage of substitutions in early and late populations in FIVC sequences from single and dual infections. (**A**) Percentage of total substitutions in each gene that are G to A substitutions. Dual late FIVC genomes have the lowest percentage of G to A substitutions. (**B**) Percentage of total substitutions in each gene that are each possible transversion. The di-nucleotide symbol indicates that the first nucleotide is replaced by the second, e.g., GT indicates a G to T substitution. Data are shown for three genes; ui is the 3′ end of the pol gene encoding dUTPase and integrase.

Increase in the value of κ could be due to an overall increase in transitions or a decrease in transversions. We evaluated the nature of the substitution bias in dual late FIVC sequences because our previous studies on PLV evolution in cats demonstrated a significant impact of cytidine deaminase activity on viral genomes concomitant with an increased rate of G to A substitutions [24]. Analysis revealed two distinct phenomena. (1) A single clone from dual infected cat 4196 at day 17 post-FIVC infection was confirmed to be G to A hypermutated using the hypermut 2 and HperPack [25] programs. Approximately 155 of 175 mutations in the 4604 bp sequence were G to A substitutions (p < 0.000001) (Figure 3). (2) The pattern of nucleotide substitutions in the remaining non-hypermutated sequences was evaluated using phylogeny-based simulation as described in the Materials and Methods section. OrfA was excluded because it contained few nucleotide polymorphisms. Although the total number of T to C substitutions was higher in *vif* in dual late genomes *versus* the rest of the groups, transitions were not overrepresented in FIVC from dual infected cats (Figure S1). In fact, G to A substitutions were lowest in the dual late group (Figure 4A). With the exception of A to T substitutions, the percentage of all other possible transversions in dual late FIVC sequences was lower than in other groups (Figure 4B). These results indicate that the increase in κ in FIVC from dual early to dual late was due to a decline in transversions, not an increase in transitions. By contrast, the percentage of each transversion typically increased from early to late groups in single infections, which was reflected in a marginal decrease in κ (Table 3).

### 2.4. Discussion

**Figure 5:**
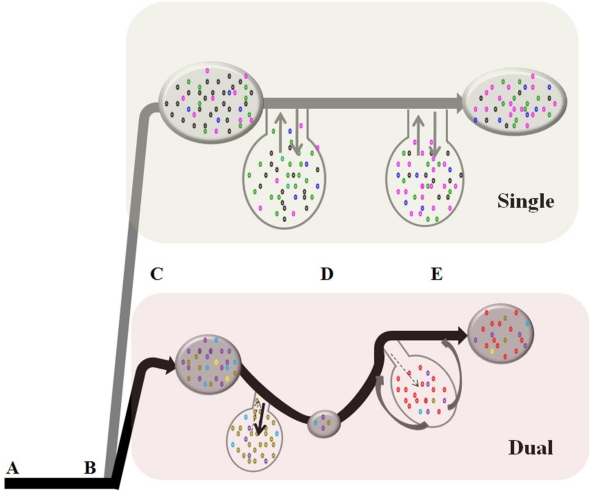
Schematic model of the dynamics of FIVC in single and dual infection. Key elements of FIVC infection dynamics in dual infected cats are indicated with letters. **A**: Initial infection of 10 cats with PLV or sham inoculated. **B**: (28 days post PLV infection); Infection of five cats in each group with FIVC (4 groups total). **C**: Peak effective population size one week after FIVC infection. **D**: Population bottleneck between week 3–7 in dual infected cats. **E**: FIVC recovery from bottleneck. The black and grey lines represent changes in FIVC effective population size from dual and single infected cats, respectively. The circles along the lines are proportional to FIVC population size in PBMC and circles adjacent to the line represent hypothetical dynamics of virus infection in peripheral tissues. The number and color of dots in each tissue ‘circle’ represent genetic diversity in each FIVC population. Possible mechanisms for the bottleneck related to changes in infected cell dynamics include decreased immigration of infected cells from peripheral tissues to blood (dashed arrow) or increased emigration from peripheral blood to tissues (bold arrow). The recovered population (**E**) in dual infected cats has unique substitution features (see text) suggesting that the majority of infected cells sampled in peripheral blood might be coming from a tissue compartment or cell type not represented in the pre-bottleneck sample (indicated by the curved arrows). An alternative explanation is that the recovered population derives from a genetically impoverished population depicted at **D** that survived the bottleneck. The population size of FIVC in single infection is stable throughout this period (**C**–**E**).

We have previously demonstrated that a primary infection with apathogenic PLV prevented the onset of CD4 decline induced by a subsequent infection with pathogenic FIVC when challenge occurred 28 days after PLV infection. PLV infection did not significantly alter FIVC proviral or plasma load and did not induce FIVC specific serum neutralizing antibodies [4]. In the present study, we investigated discernable effects of PLV infection on FIVC population dynamics using population genetics approaches. Our data indicate that, despite low levels of replicating PLV, there is a profound and prolonged effect of PLV on FIVC infection. A model summarizing these impacts is presented in Figure 5 and discussed below.

In the week following challenge, FIVC populations in both single and dual infected cats rapidly expanded. However, in dual infected cats, the effective population size of FIVC was significantly smaller than for FIVC in the single infection environment. The lower FIVC population size in dual infected cats could be a direct consequence of a decrease in available susceptible target cells by PLV. However, there are no data to indicate that availability of susceptible cells is a limiting factor for FIVC infection in general, and in our study, there were no significant differences in blood counts of naïve and PLV-infected cats [4]. Although we did not detect anti-FIVC neutralization titers or cell mediated immune parameters [4], it is possible that PLV-generated effective humoral or cellular immunity, which was below our limits of detection. More detailed evaluation is required to rule out this possibility. A third consideration is that prior PLV infection resulted in a host environment with residual or primed target cells poised to raise a more effective innate response, which limited the ability of FIVC to infect or replicate in some cells. This is an intriguing possibility in light of our previous observations implicating cytidine deaminase editing during PLV infection [24]. Our finding of one hypermutated FIVC clone from a dual infected cat at the onset of the bottleneck and the significant decline of G to A substitutions in the recovered population suggests that cytidine deaminase activity could be involved in the reduction of FIVC population size in dual infection; this possibility merits more thorough investigation.

After the expansion phase, the FIVC effective population size in the dual infection environment was stable for two weeks followed by a sharp population decline. There are several plausible mechanisms to account for the FIVC bottleneck. The immunocyte population that supports productive FIVC infection could be markedly reduced in the face of persistent PLV activation, although as noted above, cell counts and phenotypes were similar in dual and single infected animals. We note that our samples come from provirus in circulating PBMC representing a mix of infected cells moving into and out of the blood from various tissues. Tissue compartmentalization of lentiviruses is well documented [26–37]. Thus, without evoking differences in individual cell infection or production rates, changes in migration patterns of infected PBMC from blood to tissue could lead to the difference in effective FIVC population size in dual and single infections. For example, either sequestration of infected PBMC in a solid tissue, such as lymph node or bone marrow, or increased emigration of infected cells out of the circulation to sites of inflammation in dual infected cats are consistent with our results. It is noteworthy that near day 24 post-FIVC infection, dual infected cats developed a distinct immunological profile in PBMC, which consisted of elevated numbers of CD8 and CD25 cells and higher expression of IL4, FAS, and IFNγ [38]. The bottleneck corresponded temporally to the development of this pro-inflammatory immunological profile [38], and to transient restriction of proviral load [4], in dual infected cat PBMC. This is the only time point at which there is sufficient signal in the census population size to reflect the decrease in effective population size, which suggests that many provirus containing cells are not productively infected. Cumulatively, these data support that a unique immunological environment, which could be tissue specific but is transiently reflected peripherally, in dual infected cats contributed to the FIVC population decline.

Restriction in effective population size by any mechanism discussed above will increase the impact of genetic drift because there is a stronger effect of drift on populations with lower effective population size. We observed both lower effective population size and higher mutation rate of FIVC in dual infection environment, suggesting that drift might have contributed to the observed bottleneck. In a viral population with smaller effective population size, accelerated rates of mutation can also be caused by positive selection, which tends to rapidly fix beneficial mutations in the population. However, we found no evidence of positive selection pressures in either single or dual infection environments. Accumulation of deleterious mutations is another consequence of drift on small populations. This effect, called Muller’s ratchet [12], can lead to a genetic bottleneck due to loss of fitness and has been reported for HIV-1 [19–21] and for several other RNA viruses [13–18]. The gradual accumulation of deleterious mutations due to drift could be accelerated by cytidine deaminase editing of viral genomes, which renders many genomes defective [24].

FIVC populations in dual infected cats recovered to initial levels approximately seven weeks post FIVC infection and remained constant and consistently lower than FIVC in single infected cats for the duration of the experiment. Populations recovering from a bottleneck could have decreased fitness because most of the genetic variation had been eliminated. Thus, the nature of the population that reestablished the FIVC population is important for understanding long term clinical consequences of dual infection. We used population genetics approaches to determine how the late populations compared to those that established infections in dual and single infected cats. The AMOVA results demonstrated that there was temporal structure in both infection environments. Temporal population structure, which is the sequential replacement of virus genotypes over time, has been described for HIV-1 infections in blood and tissues [32], and is expected given the mobility patterns of circulating T cells. However, based on F_ST_ analyses, temporal structure was more pronounced in the dual infection environment. These data are consistent with the hypothesis that the recovered population derived from a cell type that is poorly represented in single infected cats and at early time points. The specific pattern of substitutions was also revealing of differences in temporal population structure that occurred in the presence or absence of a bottleneck. Transversions are more likely to cause non-synonymous changes that accumulate as deleterious substitutions are purged and the population evolves to greater fitness. If the late population is descendant from the early population and did not experience a genetic bottleneck, one would expect a higher number of transversions in the late population. This was, in fact, noted for FIVC genomes from single infected cats; the value of κ for FIVC in the early population was higher than that of the late population. However, there was an unusually high value of κ in dual late compared to dual early FIVC populations. We show that this was due to a decrease in the number of transversions rather than an increase in transitions. This is consistent with the premise that proviral genomes emerging from the bottleneck have lower fitness; they carry an excess of deleterious mutations, which have not yet been purged from the population [19].

The dual late FIVC population had a significantly lower number of G to A substitutions compared to any of the other populations. An excess of G to A substitutions can occur through enzymatic editing mechanisms [24,39–41] which was evident from the single clone from a dual infected cat (Figure 3), or through misincorporation of dUTP [42]. The lentivirus accessory gene *vif* protects against cytidine deamination of the viral genome [40,43]. Cell type specific expression of human cytidine deaminases, APOBECs, has been demonstrated in humans; additionally, regulation of APOBECs expression by innate effectors is also tissue specific [44]. FIV also encodes a dUTPase, which decreases the incorporation of uracil into the newly synthesized viral DNA. Defects in viral dUTPase, which increase overall mutation rate, can be overcome in actively replicating cells, which express high levels of cellular dUTPase [45]. Our findings of a low level of G to A substitutions are consistent with the data on tissue specific expression and regulation of host restriction factors responsible for this substitution bias. Thus, FIVC-infected PBMC sampled in the blood of dual infected cats may be derived from a tissue compartment or cell type where host restriction is relaxed. The lower effective population size after recovery from the bottleneck would indicate that this site is not optimal for FIVC replication.

Our data cumulatively support a model for long term, and potentially cell or tissue specific, innate immune priming that substantially alters FIVC genomic population structure following PLV infection. This work significantly contributes towards understanding the mechanism by which attenuated viruses control disease caused by more virulent strains in the absence of known adaptive immune responses. If confirmed by additional experiments, this mechanism could elucidate a sustained phenotype for innate immunity that is instrumental in lentivirus control and lead to novel therapeutic avenues.

## 3. Materials and methods

### 3.1. Experimental Design, Cloning, and Sequencing

Detail on the experimental design was reported in Terwee *et al*. [4]. Briefly, two groups of 10 cats each were inoculated with PLV or sham inoculated at day 0. At day 28, five cats in each group were inoculated intravenously with the molecular clone FIVC36 (FIVC hereafter) [3]. Blood samples from day 17, 52, 80/87 post-FIVC challenge (days 45, 80, 108/115 post-PLV) were used to obtain FIVC sequence data. All animal work was conducted under approval of the Colorado State University Animal Care and Use Committee.

Sequencing of a 4604 bp of the 3′ end of the FIVC genome from four cats in the PLV + FIVC group (dual infected) and four cats in the FIVC only group (single infected) was carried out using previously described protocols [24]. The primers for nested PCR amplification were 3FIVC5F (5′CTGCCAGGAGAAGTAAAAGTAA) and 3FIVC6R (CTCAAAGGGAAGAAATCAGC). First and second round amplification conditions were 35 cycles of 95 °C denaturation (30 s), 52 °C anneal (30 s), 72 °C extension (5 min). Second round primers contained NotI and BamHI restriction sites and conditions were 3FIVC7NotF (CCTGCGGCCGCTTGGGGATTGATACTTG) and 3FIVC8BamR (CGGAGGATCCACATTGCCTACCATTTCT). An annealing temperature of 54 °C was used for second round PCR. Products were gel purified, digested with the appropriate enzyme and cloned into a Not1 and BamH1 digested pBS vector. For each cat, 7 to 8 clones derived from different first round PCR were sequenced. DNASTAR software package (DNASTAR Inc. WI, USA) was used for sequence assembly, editing, and alignment. The inoculating sequence (AY600517) was used as the reference sequence for alignment. A total of 151 sequences (dual: 78 and single: 74), which included four coding genes (dUTPase and integrase region of pol (ui), vif, orfA, and env) were obtained. One clone from day 17 of a dual infected cat (4196) was hypermutated and was excluded from the population genetic analyses.

### 3.2. Estimates of Genetic Diversities and Population Dynamics

We took two approaches to characterize the evolutionary genetics of FIVC in the dual and single infection environments. Our first analyses employed distance-based summary statistics [46,47], which are independent of time. This approach determines differences in genetic diversities between two viral populations arising from variation in effective population size, differential selection pressures, recombination events, and migration among tissue compartments. The two indices of genetic diversity estimated here are θw [47] and θπ [46]. The parameter θw is based on the number of segregating (polymorphic) sites in the data set; the parameter θπ is based on pairwise differences. Tajima’s D was used to determine the intensity of selection pressure and to explore the population history of each viral population [48]. The genetic diversity indices were estimated using the DNAsp version 5 [49].

While population history may be inferred using statistics such as Tajima’s D [48] and Fu’s Fs [50], unique events that occurred in the past will not be revealed with these estimates because they are independent of time. A Bayesian coalescent approach is more appropriate to infer past changes in population size from serial sampled data collected from a population [22,23], which make no prior assumptions about population history. Therefore, our second approach was to perform Bayesian skyline plot (BSP) analyses using BEAST version 1.4.8 [22] to infer the dynamics of FIVC in single and dual infection environments. The HKY + G was the best-fit nucleotide substitution model for each data set selected by the hierarchical likelihood ratio tests (hLRTs) as implemented in the Modeltest version 3.7 [51]. A simple HKY model of nucleotide substitution, both with the strict molecular clock and with the relaxed clock under uncorrelated log-normal distribution (UCLD) was used to avoid over-parameterization due to the limited diversity and sampling time range with our data. The coefficient of co-variation (CoV) inferred from the relaxed model of the respective group was used as an indicator of the extent of rate heterogeneities in each viral lineage. If the 95% HPD of CoV encompasses zero, then clock-like behavior cannot be rejected. MCMC chains were run for a total of 100 million generations and 1% burnin was discarded from each chain. MCMC chains were run multiple times and the convergence was checked using Tracer 1.4 [52]. Since all clones were derived within the 87 days after challenge with FIVC, the TMRCA was constrained to 90 days. The uncertainty in the data is reflected in the 95% HPD intervals. The BSP [23] provided estimates of effective population size, in terms of relative genetic diversity, for virus populations replicating in single and dual infection environments.

### 3.3. Analyses of Temporal Population Structure

To examine the temporal population structuring, we partitioned the data into four groups: single infection-early (SE), single infection-late (SL), dual infection-early (DE), and dual infection-late (DL). The early time point referred to the samples collected at day-17, and late time point referred to the samples collected at day-80/day-87 post FIVC infection. Analyses of molecular variance (AMOVA) were carried out using the Arlequin version 3.1 [53] to evaluate how the genetic variances in these four infection groups are partitioned. This is a genetic distance-based test that first estimates pairwise distances within and between predefined groups and then partitions the covariance components to the respective groups. The total molecular variance can be divided into the covariance component due to: (a) differences among individuals within single and within dual infection environment (F_ST_), (b) a covariance component due to differences between early and late populations within single and within dual infection environments (F_SC_), and (c) a covariance component due to differences between single and dual infection environments (F_CT_). The significance of the covariance components is tested using non-parametric permutation procedures [54]. Statistical support is determined non-parametrically by permuting group assignments 10,000 times. The pair-wise genetic distances measured as F_ST_ [55] among the four groups were also estimated using Arlequin and their significance were determined non-parametrically with 10,000 simulations. The F_ST_ matrix was used to reconstruct the neighbor joining tree using the NEIGHBOR program as implemented in PHYLIP version 3.69 [56].

### 3.4. Pattern of Nucleotide Substitutions

#### 3.4.1. Selection Analyses

We performed codon specific selection analyses on each protein coding gene (ui, vif, orfA, and env) using the maximum likelihood based codon substitution model implemented in CODEML of PAML version 4.1 [57], and Fixed Effects Likelihood (FEL) and Single Likelihood and Ancestor Counting (SLAC) methods implemented in the datamonkey.org web server [58]. To avoid the possibility of false positives, we considered a codon to be under positive selection if that site was identified by both methods. The transition: transversion rate ratio (Kappa, κ) for each gene at each time point was estimated using CODEML.

#### 3.4.2. Substitution Bias

Prior to further genetic analyses, all the sequences were analyzed for hypermutation by the cytidine deaminase editing mechanism using the Hypermut 2 [59] and HyperPack [25] software using the inoculating sequence (AY600517) as the parental sequence.

Alignments were developed from 3′-genome FIVC sequences obtained for clones sequenced from PBMC either early (day 17) or late (day 80 or 87) post-infection. Separate alignments were established for ui, vif, OrfA, and env genes. The sequence for the corresponding gene region in AY600517 was included in each alignment as an outgroup. Further, larger (“combined”) alignments were developed by including all of the sequences available for each combination of time (early, late) and infection state (single, dual). The substitutions occurring in each evolutionary history were characterized by phylogenetic analysis. A phylogenetic tree was estimated for each sequence alignment using PAUP* [60] with the GTR + G model of evolution. The tree then formed the basis for estimating the occurrence of nucleotide substitutions; BASEML, a part of PAML v4.1 [57], was used to infer the ancestral states at each internal node of the tree. We assessed whether the substitutions occurred randomly with respect to local sequence context by comparing the observed results with those obtained by random sequence evolution. The evolution of sequences at random with respect to local context was simulated in Seq-Gen v1.3.2 [61], using the inferred phylogenetic tree and the parameters inferred from it. Simulated sequence evolution was performed for 100 replicates for each evolutionary history. For each alignment and type of substitution, the relative frequency of each di-nucleotide context was determined. From the 100 replicates, the distributions of these relative frequencies were determined. An observed context was judged to be significantly under-represented if it fell below the 5 or 1 percentiles of the distribution, or over-represented if it were above the 95 or 99 percentiles. The four treatment effects (single early, single late, dual early, dual late) were investigated using the combined sequence alignments.

## Figures and Tables

**Table 1:** Mutation rates of FIV genome in single and dual infected cats.

**Group**	**Clock model**	**Mutation rate (×10^−5^/site/day)**	CoV*
Single	Strict	10.60 (9.23–12.5)	N.A
	Relaxed	4.60 (4.21–5.00)	0.866 (0.698–1.057)
Dual	Strict	11.3 (9.87–12.9)	N.A
	Relaxed	6.94 (5.27–8.12)	0.884 (0.681–1.116)

Estimates are based on the HKY model with uncorrelated log-normal distribution (UCLD) and Time to the Most Recent Common Ancestor (TMRCA) was constrained (90 days);

* CoV: Coefficient of Variation. N.A: Not applicable.

**Table 2:** Analyses of Molecular Variance (AMOVA) showing the hierarchical genetic partitioning in FIV from single and dual infections.

**Source of variation**	**d.f.**	**Sum of squares**	**Variance components**	**Percentage of variation**	**Fixation index**
Among groups	1	11.208	−0.01690 Va	−0.14	F_CT_: −0.00175
Among populations within groups	2	24.364	0.08847 Vb	0.92	*F_SC_: 0.00915
Within populations	114	1092.68	9.58492 Vc	99.26	*F_ST_: 0.00741
Total	117	1128.25	9.65649		

The data was partitioned into four temporally-spaced populations: single early (SE), single late (SL), dual early (DE) and dual late (DL). Single group include SE and SL, and dual group include DE and DL; Va, Vb, and Vc are the associate covariance components. F_CT_, F_ST_, and F_SC_ are the F-statistics;

* Significant values at P < 0.001 after 10,000 permutations.

**Table 3:** Transition and transversion rate ratio (kappa, k) for ui, vif, and env genes at early and late time point of single and dual infection groups.

	**ui**		**vif**		**env**	
		
Kappa (k = Ts/Tv)	Early	Late	Early	Late	Early	Late
			
Single	17.1712	10.2651	9.0782	7.88854	14.8736	7.88854
						
Dual	8.868	20.1281	10.2285	33.0202	13.6558	26.5212

Horizontal dark grey shading shows the trend from early to late for each gene of the respective infection group. The vertical light grey shading shows the trend in late phase.
